# Carbon dioxide stimulates lake primary production

**DOI:** 10.1038/s41598-018-29166-3

**Published:** 2018-07-18

**Authors:** Mohammed Hamdan, Pär Byström, Erin R. Hotchkiss, Mohammed J. Al-Haidarey, Jenny Ask, Jan Karlsson

**Affiliations:** 1 Department of Ecology and Environmental Science, Umeå University, 90187 Umeå, Sweden; 2 Department of Biological Sciences, Virginia Polytechnic Institute and State University, Blacksburg, VA 24061 USA

**Keywords:** Ecology, Biogeochemistry

## Abstract

Gross primary production (GPP) is a fundamental ecosystem process that sequesters carbon dioxide (CO_2_) and forms the resource base for higher trophic levels. Still, the relative contribution of different controls on GPP at the whole-ecosystem scale is far from resolved. Here we show, by manipulating CO_2_ concentrations in large-scale experimental pond ecosystems, that CO_2_ availability is a key driver of whole-ecosystem GPP. This result suggests we need to reformulate past conceptual models describing controls of lake ecosystem productivity and include our findings when developing models used to predict future lake ecosystem responses to environmental change.

## Introduction

The rate at which primary producers fix inorganic carbon controls the supply of organic matter to food webs and influences the biogeochemistry of aquatic ecosystems^1,2^. The role of inorganic nutrients and light in controlling GPP is indisputable and has been extensively studied^3–5^.

Data also suggest that CO_2_ availability can constrain GPP. Small scale experimental studies, and comparative studies of lakes, have shown that elevated CO_2_ concentration promotes phytoplankton biomass and GPP^6,7^. Similarly, CO_2_ concentration can limit phytoplankton growth in marine ecosystems^8^. However, aquatic ecosystems are comprised of both pelagic and benthic habitats, and recent studies emphasize the importance to include both habitats to understand whole-ecosystem productivity and function^3,4^. Presently, the role of CO_2_ availability for whole-ecosystem GPP, especially in shallow lakes where both pelagic and benthic GPP may contribute significantly to whole-ecosystem GPP, has never been tested^9^. Improved knowledge of the control of GPP is fundamental for understanding ecosystem function and impacts of environmental change.

Here we carried out a large-scale experiment to test the role of CO_2_ availability for whole-ecosystem GPP. We used a novel approach where part of the CO_2_ that accumulated under ice cover over winter was released to the atmosphere by manipulation of the spring ice cover. The aim was to decrease CO_2_ concentrations in the water column while having a limited effect on light, temperature, and other key environmental factors. In spring, the ice cover was decreased twice, by first removing 10% and then 50%, to impose a gradual but drastic difference in CO_2_ concentrations in the treatment ponds. Whole-ecosystem GPP was estimated using dissolved oxygen time series data, and potential abiotic and biotic drivers of GPP were monitored over the course of the experiment.

## Results and Discussion

**Figure 1:**
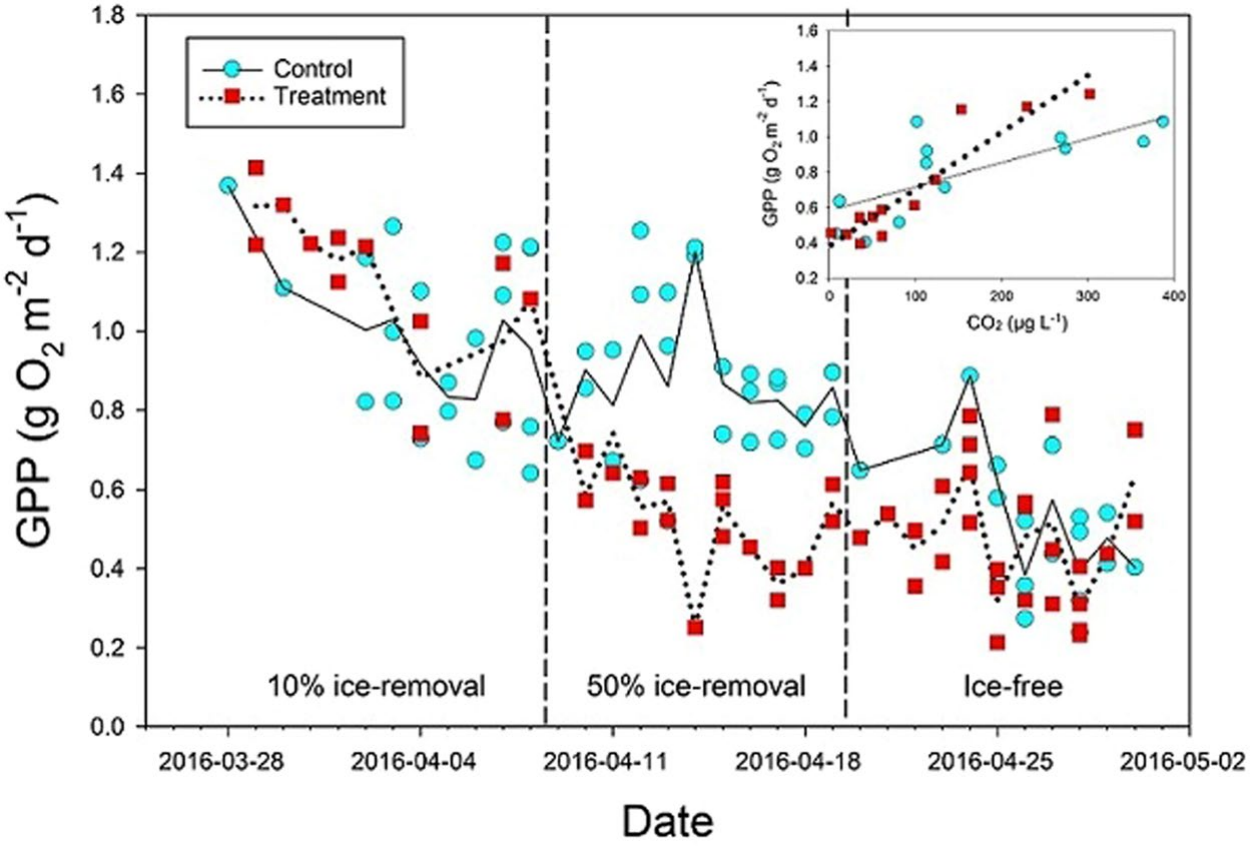
Daily gross primary production (GPP) and correlations between GPP and carbon dioxide (CO_2_) concentrations (inserted) in control (full line) and treatment (dashed line) ponds. The trend lines are moving averages of daily GPP.

**Figure 2:**
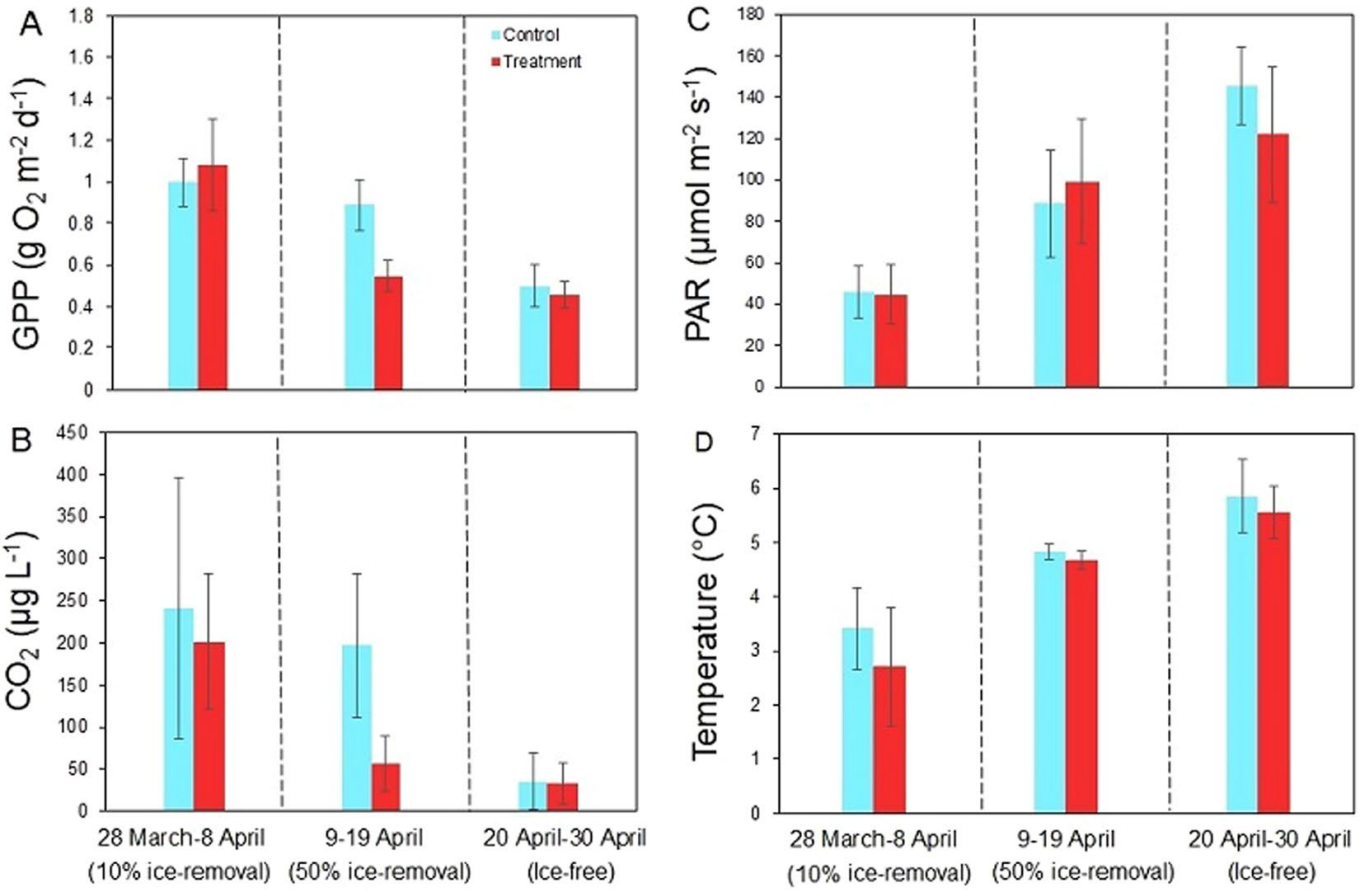
Average (±1 SD) gross primary production (GPP) (**A**), carbon dioxide (CO_2_) (**B**), photosynthetically active radiation (PAR) (**C**) and temperature (**D**) for the different periods during the experiment.

The GPP and CO_2_ concentration in control ponds were relatively high under ice compared to after ice break-up in spring (Figs 1, 2). The 10% ice-removal treatment did not change GPP or decrease CO_2_ concentration compared to control ponds (Fig. 2 and Table 1). In contrast, the 50% ice-removal treatment decreased both GPP and CO_2_ compared to control ponds (Fig. 2 and Table 1). Finally, after ice break-up, there were no differences in GPP and CO_2_ between control and treatment ponds (Figs 1, 2 and Table 1). There was a positive correlation between GPP and CO_2_ concentration in both control and treatment ponds (Fig. 1 and Table 1). These results show that the CO_2_ concentration was a key controlling factor for GPP in the ponds.

**Table 1:** Statistical Analysis results.

Test	Ponds	Treatment	Variables	Statistics	*p*-values	n	*df*
**T-test**				***t-*value**			
	C vs. T	10%	GPP	−0.77	0.49	4	3
	C vs. T	10%	CO_2_	0.49	0.56	4	3
	C vs. T	10%	PAR	0.23	0.82	4	3
	C vs. T	10%	Temp.	2.6	0.07	4	3
	C vs. T	50%	GPP	12.21	<0.01	4	3
	C vs. T	50%	CO_2_	3.64	<0.05	4	3
	C vs. T	50%	PAR	−1.30	0.28	4	3
	C vs. T	50%	Temp.	0.97	0.40	4	3
	C vs. T	Ice-free	GPP	1.18	0.32	4	3
	C vs. T	Ice-free	CO_2_	0.13	0.90	4	3
	C vs. T	Ice-free	PAR	0.92	0.42	4	3
	C vs. T	Ice-free	Temp.	0.80	0.47	4	3
**Pearson’s** ***r***				***r*-value**			
	C		GPP vs. CO_2_	0.73	<0.01		
	T		GPP vs. CO_2_	0.93	<0.001		
	C		GPP vs. PAR	−0.86	<0.001		
	T		GPP vs. PAR	−0.69	<0.05		
	C		GPP vs. Temp.	−0.80	<0.01		
	T		GPP vs. Temp.	−0.73	<0.01		
**ANOVA**				***F*-value**			
	C vs. T		GPP * time	6.92	<0.05		2, 12
	C vs. T		CO_2_ * time	3.95	<0.05		2, 12
	C vs. T		PAR * time	2.16	0.15		2, 12
	C vs. T		Temp.* time	0.3	0.74		2, 12

Control ponds, treatment ponds, number of replicates, degrees of freedom, 10% ice-cover removing treatment, 50% ice-cover removing treatment, Ice-free period, and linear correlation are abbreviated as C, T, n, *df*, 10%, 50%, Ice-free and *r*, respectively.

No other abiotic or biotic factors explain the patterns in GPP over time and between treatments. There were no differences in nutrient concentrations (NO_3_^−^, PO_4_^3−^ and NH_4_^+^, Supplementary Table S1), photosynthetically active radiation (PAR) or water temperature between control and treatment ponds (Fig. 2 and Table 1). Interestingly, GPP in control and treatment ponds was negatively correlated to both PAR and water temperature (Table 1). The consumer biomass (zooplankton and zoobenthos) did not differ between control and treatment ponds (Supplementary Table S1), suggesting that top-down effects on primary producers, if any, were similar and therefore should have no effect on the main patterns in GPP observed between treatment and control. As expected, pH of the water increased with decreasing CO_2_ concentrations in the ponds (Supplementary Table S1). The change in pH and carbonate system is part of the change associated with changes in CO_2_ saturation and cannot be easily separated from each other. Yet, previous studies on the effect of CO_2_ on GPP have not found any effect of pH on GPP and attributed GPP responses to CO_2_ availability and not to pH *per se*^6,7,10^.

This is the first experimental study on the role of CO_2_ in controlling whole-ecosystem GPP. Although CO_2_ has a key role as substrate for photosynthetic enzymes^11^, the CO_2_ supply is generally regarded as sufficient to meet the primary producer’s demands and that other factors are limiting photosynthetic rates. However, CO_2_ may often be at suboptimal levels for maximum photosynthetic efficiency^7^. Previous work has shown positive effects of CO_2_ on pelagic GPP^6,7^, but here we show that this effect applies also at the whole-ecosystem scale. This is important since lake habitats are not isolated units and both benthic and pelagic habitats are important for understanding whole-lake ecosystem food web dynamics and productivity^3,4^.

CO_2_ limitation effect on lake productivity is likely a general characteristic that needs to be taken into account in food web models for lakes. The CO_2_ concentrations of lakes vary largely across systems but also within systems, both spatially and temporally^12^, implying that CO_2_ availability can help to explain GPP at multiple scales. Given that lake CO_2_ supersaturation is common, including in abundant northern lakes^13^, we suggest that CO_2_ stimulation of GPP is a common but often overlooked phenomenon.

Although not explicitly studied in this experiment, these results also add important insight to the role of terrestrial organic matter for lake GPP. Export of dissolved organic matter from land is regarded to have two main effects on GPP in recipient lake ecosystems: (i) stimulating GPP by supplying nutrients^14^ and (ii) repressing GPP by supplying light absorbing substances^4^. Terrestrial organic matter is also mineralized in lakes, resulting in supersaturation of CO_2_^15,16^, and our results suggest this will stimulate GPP. It is likely that CO_2_ stimulates GPP in ecosystems with low to moderate terrestrial organic matter inputs, up to a point when light becomes suboptimal, after which further increase in terrestrial organic matter inputs will have an overall negative effect on GPP due to light limitation. Future research should assess the role of CO_2_ for GPP across various type of lakes.

The results from this study provide fundamental knowledge of the effects of CO_2_ dynamics on biomass production in lake food webs. More broadly, these results have major implications for a greater understanding of the effects of climate change on lake productivity, as CO_2_ dynamics in lakes are controlled by climate-dependent factors such as terrestrial carbon export, hydrologic residence time, metabolic process rates, mixing regimes and ice conditions^12,17,18^. We stress the need for future research efforts, where the effects of CO_2_ are incorporated in both experiments and models of lake ecosystem function to generate patterns at the whole-ecosystem scale.

## Methods

### Study site

The study was performed in the Umeå University Experimental Ecosystem Facility (EXEF) in northern Sweden (63°48′N, 20°14′E). The pond is divided into 20 enclosures (12.5 * 7.3 m, average depth 1.48 m) by thick non-permeable tarpaulins and each pond has a 7.3 m long natural shoreline and a bottom substrate of soft sediment. Each pond has a natural food web including basal producers (algae and bacteria), consumers (insect larvae and zooplankton) and a reproducing top-consumer population (nine-spine Sticklebacks), where benthic GPP constitutes approximately 50% of whole-ecosystem GPP^19^. For further details see^19,20^. In this study we used eight enclosures: we manipulated the CO_2_ concentration in four enclosures by ice cover removal (treatment), and four enclosures served as controls. Ice formed naturally on the ponds in mid-November 2015, and the experiment was carried out in March-April 2016. The ice cover removal was preformed twice in 2016 on the treatment ponds; 10% removal from 28^th^ March to 8^th^ April and 50% from 9^th^ to 19^th^ April, i.e. ten days for each treatment. The four control ponds experienced a natural and gradual ice melting. The treatment and control ponds became ice-free between 20^th^ of April and 1^st^ of May. The ice-removal treatment was executed by making vertical bores in the ice cover with an ice drill. Pieces of ice were cut out in-between the bores by a large-ice saw and then pushed in under the remaining ice.

### Data collection

CO_2_ concentrations were estimated every second day by using a headspace equilibration technique^21^ and analysis by gas chromatograph (Perkin Elmer Clarus 500). At the same time, nutrients (NO_3_^−^, PO_4_^3−^, and NH_4_^+^) were sampled by filtering water through burnt (550 °C, 4 h) 0.45 μm GF/F filters to 50 ml Falcon tubes and stored in the freezer until analyzed with photometric flow injection analysis (FIA) methods^22^.

Dissolved oxygen (DO) and water temperature were measured during the experiment period at ten-minute intervals by logging sensors (MiniDOT, PME, Vista, CA, USA) which were deployed at 0.5 m below the water surface in the center of each pond. Photosynthetic available radiation (PAR) was measured at ten-minute intervals by light sensors (SQ-110, Apogee USA) deployed at 0.8 m below the water surface in the center of each pond. Temperature and PAR data were converted to daily means for each enclosure based on 144 measures (6 per hour × 24 hours) and then a mean for all days within each treatment period during the experiment and finally we got a mean for each group (control and treatment) that contains four enclosures. Wind speed was recorded every ten minutes by a climate station next to the pond.

### Whole-ecosystem gross primary production (GPP) estimates

From the oxygen sensor data whole-ecosystem GPP, integrating GPP in both benthic and pelagic habitats in these non-stratified ponds, was calculated with inverse modeling and Bayesian parameter estimation using a similar parameter estimation approach as for diel DO in streams^23^, but modified for pond ecosystems “equation (1)”:

1 $$m{O}_{i}=m{O}_{i-1}+(\frac{GPP}{zmix}\ast \frac{PA{R}_{i}}{PAR})+(\frac{ER}{zmix}\ast {\rm{\Delta }}t)+{K}_{i}({O}_{sat,i}-m{O}_{i-1}){\rm{\Delta }}t$$

where mOi is modeled DO at time i (g O_2_ m^−3^) given parameter estimates of GPP and ecosystem respiration (ER; g O_2_ m^−2^ d^−1^). Because changes in O_2_ are a function of GPP, ER, and gas exchange, we calculated daily air-water O_2_ fluxes based on O_2_ saturation and the temperature-corrected gas exchange velocity for O_2_ (*Ki*, d^−1^). *Ki* was estimated from K600 derived from wind speed^24^. The emission flux of O_2_ (${K}_{i}({O}_{sat,i}-m{O}_{i-1}){\rm{\Delta }}t$) was corrected for changes in pond area open to the atmosphere (0–100% with changing ice cover) and zmix (mean of mixing depth; m), where zmix varied daily according to changes in water column depth depending on ice cover thickness. The metabolism model used a “random walk” metropolis algorithm and Markov Chain Monte Carlo (MCMC) sampling from the “metrop” function in the “mcmc” package of the statistical program R^25^ to find the best fit between measured and modelled O_2_ data given model estimates of GPP and ER. Each parameter estimate was derived from 10000 model iterations after removing an initial 1000 iterations of “burn-in” from parameter starting values. We checked for convergence of parameter estimates and removed days with negative GPP and with poor fits between measured and modeled O_2_ before assessing the response of GPP to changes in CO_2_.

### Invertebrate sampling

Zooplankton were sampled by a zooplankton net (diameter 20 cm, 100 µm mesh size) drawn vertically trough whole water column and preserved in Lugol’s solution. Zoobenthos were sampled with a net (30 cm wide, 1 mm mesh size), drawn at the bottom substrate for a distance of 1 m, and preserved in ethanol. Zooplankton and zoobenthos lengths were measured to obtain dry biomass using length-weight regressions^26,27^.

### Statistical analyses

Statistics (SPSS 20 and R v3.2.3) are based on individual pond means of measured response variables. Results were tested for time effects between treatments periods by using repeated measures ANOVA, for differences between the control and treatment within treatment periods by using standard *t-tests*, and for correlations between selected variables by using Pearson correlation coefficient.

### Data availability

The datasets generated during and/or analyzed during the current study are available from the corresponding author on request.

## Electronic supplementary material

Table S1.
