# Author Correction: Carbon dioxide stimulates lake primary production

**DOI:** 10.1038/s41598-020-67061-y

**Published:** 2020-06-12

**Authors:** Mohammed Hamdan, Pär Byström, Erin R. Hotchkiss, Mohammed J. Al-Haidarey, Jenny Ask, Jan Karlsson

**Affiliations:** 1 Department of Ecology and Environmental Science, Umeå University, 90187 Umeå, Sweden; 2 Department of Biological Sciences, Virginia Polytechnic Institute and State University, Blacksburg, VA 24061 USA

Correction to: *Scientific Reports* 10.1038/s41598-018-29166-3, published online 18 July 2018

In Figures 1 and 2B, the units for CO_2_ should be given as ‘µM’ instead of ‘µg L^−1^’. The correct Figures 1 and 2 appear below.

**Figure 1:**
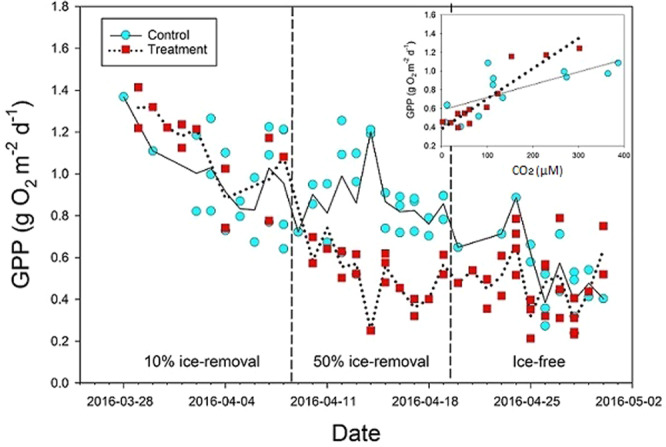
.

**Figure 2:**
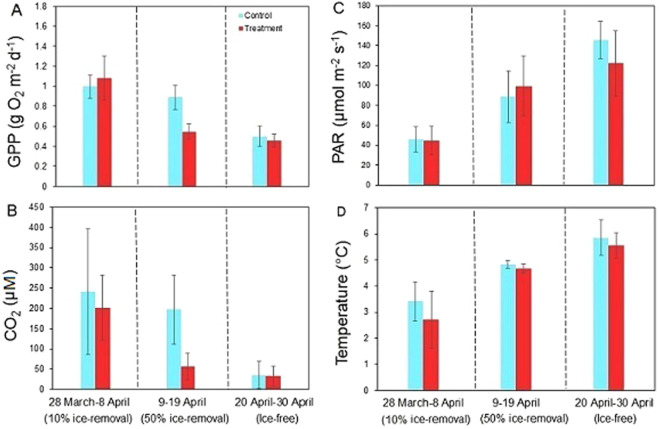
.

