# Supplementary material for: Carbon dioxide stimulates lake primary production
Source: Sci Rep. 2018 Jul 18;8:10878. doi: 10.1038/s41598-018-29166-3 (PMC6052161; doi:10.1038/s41598-018-29166-3)
Supplement: Supplementary file 1 — Table S1. [file 41598_2018_29166_MOESM1_ESM.pdf]

Carbon dioxide stimulates lake primary production

Mohammed Hamdan[1*], Pär Byström[1], Erin R. Hotchkiss[2], Mohammed J. Al-Haidarey[1], Jenny Ask[1], Jan Karlsson[1]

[1]Department of Ecology and Environmental Science, Umeå University, 90187 Umeå, Sweden.

[2]Department of Biological Sciences, Virginia Polytechnic Institute and State University, Blacksburg, VA 24061, USA.

Supplementary table

Table S1. Mean values for pH, nutrient (nitrate (NO_3^-), phosphate (PO_4^{3-}) and ammonium (NH_4^+)) concentrations (µg L^{-1}) and consumer biomass (zooplankton (Zoop., µg L^{-1}) and zoobenthos (Zoob., g m^{-2}), and the corresponding statistical results.

Test	Ponds	Treatment	Variables	Statistics	*p*-values	n	*df*	mean±1SD	mean±1SD
**T-test**				***t*-value**				**C**	**T**
	C vs. T	10%	pH	2.37	0.09	4	3	6.85±0.10	6.81±0.12
	C vs. T	10%	NO_3^-	-0.18	0.86	4	3	10.18±7.43	10.98±1.87
	C vs. T	10%	PO_4^{3-}	-0.92	0.42	4	3	1.83±0.58	3.10±3.32
	C vs. T	10%	NH_4^+	-1.33	0.27	4	3	7.71±2.38	21.02±20.30
	C vs. T	50%	pH	-7.02	<0.01	4	3	7.07±0.11	7.73±0.13
	C vs. T	50%	NO_3^-	0.18	0.86	4	3	11.04±5.86	10.82±5.86
	C vs. T	50%	PO_4^{3-}	-1.23	0.30	4	3	1.56±0.55	1.76±0.51
	C vs. T	50%	NH_4^+	-0.27	0.80	4	3	12.46±8.62	13.65±15.15
	C vs. T	50%	Zoop.	1.97	0.14	4	3	11.74±1.72	8.85±2.29
	C vs. T	50%	Zoob.	1.20	0.31	4	3	0.99± 0.29	0.68±0.30
	C vs. T	Ice-free	pH	0.50	0.65	4	3	8.12±0.08	8.10±0.04
	C vs. T	Ice-free	NO_3^-	-0.06	0.95	4	3	5.96±0.59	5.99±0.55
	C vs. T	Ice-free	PO_4^{3-}	-0.99	0.39	4	3	2.01±0.69	2.42±0.84
	C vs. T	Ice-free	NH_4^+	-1.39	0.25	4	3	4.60±0.57	5.85±2.12
**Pearson's *r***				***r*-value**					
	C		GPP vs. NO_3^-	0.34	0.27				
	T		GPP vs. NO_3^-	0.18	0.56				
	C		GPP vs. PO_4^{3-}	-0.09	0.76				
	T		GPP vs. PO_4^{3-}	-0.08	0.80				
	C		GPP vs. NH_4^+	0.22	0.49				
	T		GPP vs. NH_4^+	0.42	0.16				
**ANOVA**				***F*-value**					
	C vs. T		NO_3^- * time	0.01	0.98		2, 12		
	C vs. T		PO_4^{3-} * time	0.46	0.63		2, 12		
	C vs. T		NH_4^+ * time	0.80	0.47		2, 12		

Control ponds, treatment ponds, number of replicates, degrees of freedom, standard deviation, 10% ice-cover removing treatment, 50% ice-cover removing treatment, ice-free period and linear correlation are abbreviated as C, T, n, *df*, SD, 10%, 50%, Ice-free, and *r*, respectively.
